# Daptomycin plus ceftaroline salvage therapy for persistent *Staphylococcus epidermidis* bacteremia

**DOI:** 10.1002/ccr3.8486

**Published:** 2024-02-19

**Authors:** Hayden Zhang, Kylie Tran, Richard Lindley, Ravindra Dotel

**Affiliations:** ^1^ Department of Infectious Diseases Blacktown Hospital Sydney New South Wales Australia; ^2^ Blacktown Mount Druitt Clinical School Western Sydney University Sydney New South Wales Australia; ^3^ Pharmacy Department Blacktown Hospital Sydney New South Wales Australia; ^4^ Department of Geriatrics Blacktown Hospital Sydney New South Wales Australia; ^5^ Westmead Applied Research Centre University of Sydney Sydney New South Wales Australia; ^6^ The George Institute for Global Health Sydney New South Wales Australia

**Keywords:** bacteremia, ceftaroline, daptomycin, persistent, *Staphylococcus epidermidis*

## Abstract

Initial antibiotics for true *Staphylococcus epidermidis* bacteremia include vancomycin or linezolid, but if bacteremia persists, consideration should be made for salvage combination therapy regimes such as daptomycin with ceftaroline.

## 1 INTRODUCTION

*Staphylococcus epidermidis* is a coagulase‐negative gram‐positive bacterium that commonly colonizes the skin and mucous membranes of humans.^1^ It is frequently isolated from blood culture collection and is usually considered a contaminant species; however, its relevance in healthcare‐associated infections is becoming more recognized.^2^ It is the most common cause of nosocomial blood infections, usually associated with vascular access lines, because of its ability to form bacterial biofilms.^1^ Management of *S. epidermidis* bacteremia involves the removal of infected prosthetic material and an adequate course of effective antimicrobials.^3^ Bacteremia can occasionally be persistent, with a recent review reporting a median duration of 3 days.^4^ We present a challenging case of persistent *S. epidermidis* bacteremia over 21 days, where a novel antibiotic regimen was utilized for definitive treatment.

## 2 CASE REPORT

A gentleman in his 70s from home was admitted to a tertiary Australian hospital with several days of atraumatic bilateral knee arthritis. His background history included type 2 diabetes mellitus, stage 3b chronic kidney disease, atrial fibrillation, hypertension, and gout. Based on examination findings and his history of poor adherence to allopurinol, he was commenced on a short course of prednisone 25 mg daily for a clinically suspected polyarticular gout flare and improved significantly. His admission was prolonged due to requiring aged care home placement.

One month into admission, he became febrile with no focal signs or symptoms. Peripheral blood cultures collected during the initial septic work‐up flagged positive after 24 h in both aerobic and anerobic bottles with *S. epidermidis*. He had no audible murmurs, nor did he have any intravenous cannulas or prosthesis, and so this result was initially deemed to be clinically insignificant. He continued to have fevers while remaining hemodynamically stable over the next 3 days, and subsequent blood cultures continued to isolate *S. epidermidis* after 24 h incubation, raising concerns for true bacteremia. The isolate was methicillin resistant but susceptible to vancomycin with a minimum inhibitory concentration (MIC) of 2 mg/L, confirming sensitivity according to the European Committee of Antimicrobial Susceptibility Testing (EUCAST) breakpoint of 4 mg/L for coagulase‐negative *Staphylococci*.^5^ Intravenous vancomycin was commenced, with dosing guided by Bayesian modeling software as per consensus guidelines.^6^ His body mass index was 35 kg/m^2^, and creatinine clearance was 24 mL/min based on his ideal body weight. He was started on vancomycin 1000 mg, which was later increased to 1200 mg daily. AUC/MIC >400 mg.h/L was achieved by the fifth dose, and intermittent vancomycin was continued for 6 days. All intravenous catheters were reviewed and changed routinely as per local hospital policy. Unfortunately, he was unable to clear his bacteremia, and so he was switched to a continuous vancomycin infusion of 1300 mg daily to reduce variation in concentrations. Vancomycin remained therapeutic with an AUC/MIC of 500 mg.h/L on continuous infusion, which was given for another 7 days. Despite over 10 days of vancomycin at therapeutic levels, his daily blood cultures continued to isolate *S. epidermidis*.

Investigations were performed to look for sources of persisting bacteremia despite therapeutic levels of vancomycin. A computed tomography scan was performed, showing no focal abnormalities in his chest, abdomen, or pelvis. He underwent a transesophageal echocardiography, which revealed a heavily calcified degenerative stenotic aortic valve with Lambl's excrescences but no mobile vegetation. A positron emission tomography scan was performed to look for indolent sources of bacteremia, but it revealed no definitive findings. After almost 2 weeks into therapy, he developed superficial thrombophlebitis in an intravenous cannula site, which was thought to be contributory but not the root cause for the persistent bacteremia.

Linezolid MIC was 2 mg/L, considered sensitive according to EUCAST,^5^ and so therapy was changed to oral linezolid 600 mg twice daily after 12 days of vancomycin. Despite achieving a supratherapeutic linezolid trough level of 11.1 mg/L 48 h after initiation, with improvement in fevers and inflammatory markers, his blood cultures remained positive for *S. epidermidis* between 24 and 48 h of incubation.

**FIGURE 1:**
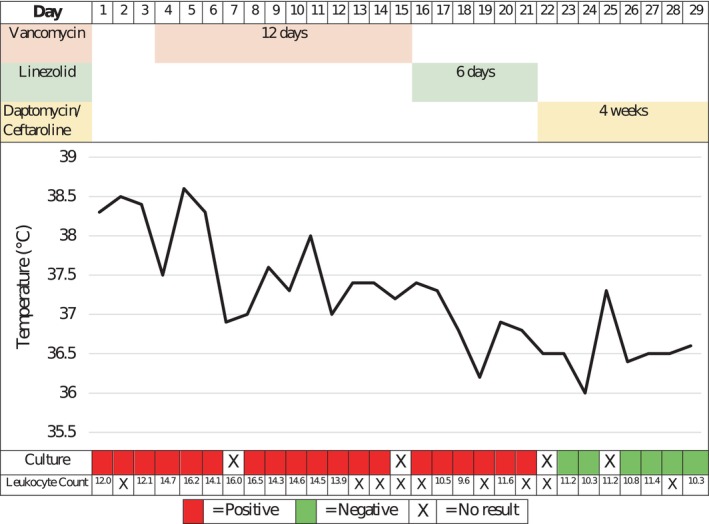
Antibiotic regime with corresponding peak temperature chart and blood culture results.

After 21 days of persistent *S. epidermidis* bacteremia (Figure 1), despite appropriately targeted antimicrobials with maintained MIC to both vancomycin and linezolid, his therapy was changed to intravenous daptomycin 8 mg/kg every 48 h with synergistic ceftaroline 300 mg three times daily, dose‐adjusted based on his estimated creatinine clearance of 24 mL/min. This decision was made based on available literature for persistent methicillin‐resistant *Staphylococcus aureus* (MRSA) bacteremias.^7^ Daptomycin and ceftaroline MICs for the isolate were 0.25 and 0.125 mg/L, respectively; the former were considered sensitive as per EUCAST,^5^ and the latter had no established breakpoint. Blood cultures taken 1 day after starting combination salvage therapy isolated no growth. He subsequently had a further five blood cultures that remained negative after 5 days of incubation. It was noted that all previous positive blood cultures had isolated the organism within 48 h, and so he was deemed to have cleared the bacteremia. Given his prolonged illness, he was treated as a complicated endovascular infection with 4 weeks of daptomycin and ceftaroline following blood culture clearance, administered via the outpatient parenteral antimicrobial therapy service, before stepping down to oral linezolid for a further 2 weeks. He remained clinically well, and surveillance blood cultures taken 4 weeks after completing antibiotic therapy remained negative.

## 3 DISCUSSION

The combination of daptomycin and ceftaroline for complicated MRSA bacteremias has been well described in retrospective studies as a salvage therapy option in persistent cases.^7^ Antimicrobial options for persistent *S. epidermidis* bacteremia are significantly lacking, particularly in the absence of a focal source. There are previous reports documenting the use of daptomycin alone with some success, including hemodialysis‐associated infective endocarditis^8^ and line‐associated neonatal sepsis.^9^ A further two cases of prosthetic valve infective endocarditis were treated with a combination of daptomycin and ceftaroline.^10^ Previous data in persistent MRSA bacteremias have suggested that heavy vancomycin exposure increases the risk of daptomycin failure,^11^ and thus lead us to decide on combination therapy. The importance of a multidisciplinary team approach in managing complex infections was also highlighted in this case, with significant contributions made by both the medical and antimicrobial stewardship pharmacy teams in decision‐making and antibiotic dose adjustment. We believe this case serves as an example of successful clearance of persistent bacteremia using this novel treatment approach as salvage therapy in the absence of any persistent focal source for *S. epidermidis*.

## AUTHOR CONTRIBUTIONS

**Hayden Zhang:** Conceptualization; data curation; investigation; writing – original draft. **Kylie Tran:** Data curation; investigation; resources; writing – review and editing. **Richard Lindley:** Conceptualization; supervision; validation; writing – review and editing. **Ravindra Dotel:** Conceptualization; data curation; investigation; supervision; writing – review and editing.

## FUNDING INFORMATION

The lead author affirms that no funding from internal or external sources was received in producing this manuscript.

## CONFLICT OF INTEREST STATEMENT

The authors declare no conflict of interest.

## ETHICS STATEMENT

Ethical approval was granted by the Western Sydney Local Health District Human Research Ethics Committee.

## CONSENT

Written informed consent was obtained from the patient to publish this report in accordance with the journal's patient consent policy.

## Data Availability

The data that support the findings of this study are available from the corresponding author upon reasonable request.
